# The influence of flint stones on a soil microbial community in the northern Negev Desert

**DOI:** 10.3934/microbiol.2017.3.580

**Published:** 2017-07-10

**Authors:** Haggai Wasserstrom, Vered Elias Ben-Ezra, Chen Sherman, Adrian Unc, Yosef Steinberger

**Affiliations:** 1 The Mina & Everard Goodman Faculty of Life Sciences, Bar-Ilan University, Ramat-Gan 5290002, Israel; 2 School of Science and the Environment, Memorial University of Newfoundland, Corner Brook, NL A2H 5G4, Canada

**Keywords:** flint stone, microbial community, functional diversity, beneath-stone microbial activity, desert environment

## Abstract

In the Negev Desert ecosystems, flint-stone cover on slopes acts as a barrier against water flow. As a result, soil moisture increases and organic matter accumulates under the stone and in the immediate surroundings, both affecting the duration of soil microbial activity. On the other hand, during the dry season (characterized by approximately 210 dew nights), flint-stone cover plays an important role in the formation of dew, which eventually trickles down beneath the stone, correspondingly enhancing biological activity. The present study examined the possible role of flint stones as hotspots for soil microbial-community activity and diversity. The results were compared with those obtained from the adjacent stone-free soils in the open spaces (OS), which served as controls. Microbial activity (respiration and biomass) and functional diversity were determined by the MicroResp™ method. In addition, estimates of genetic diversity and viable counts of bacteria and fungi [colony-forming units (CFUs)] were obtained. The soil was significantly wetter and contained more organic matter beneath the flint stones (BFS). As hypothesized, biological activity was enhanced under the stones, as described by CO_2_ evolution, microbial-community biomass functional diversity, and fungal phylogenetic diversity. BFS environments favored a greater range of catabolic functions. Taxa generally known for their stress resilience were found in the OS habitats. The results of this study elucidate the importance of flint-stone cover to soil microbial biomass, community activity, and functional diversity in the northern Negev Desert.

## 1. Introduction

The Israeli desert habitat is mostly characterized by low (<100 mm per year) and unpredictable precipitation [1], extreme daily and annual temperature variability, high radiation, and excessive evaporation. The desert soil is rough-textured, salty, and poor in organic matter. As a result, the depth of wet soil is usually shallow, with the deep layers constantly dry. Accordingly, Noy-Meir [2] defined the desert ecosystem as an environment in which the dominant limiting factor for biological activity is water availability. Any source of moisture will trigger activity and, thus, govern the functionality of soil biota.

According to Lahav and Steinberger [3], stone cover on slopes acts as a barrier, preventing water runoff and nutrient flow. Organic-matter accumulation is associated with greater catabolic activity, mineralization, and thus the availability of nutrients to secondary producers. In the Negev, hypolithic algae can be found on the soil-facing surface of stones lying on the ground. This phenomenon has been reported for over 60 years for areas around the world with extreme arid conditions [4]. The algae can be found in soil crusts and under various types of stones (i.e., flint, lime, granite, and sandstones). According to Berner and Evenari [5], flint stone does not absorb water or minerals but allows limited entry of the light necessary for photosynthesis. The hypolithic algae sustain an entire food chain composed of bacteria, fungi, protozoa, nematodes, and arthropods [6] that cannot be sustained in the exposed desert areas. Under extreme stress, the soil microbial community tends to respond with extreme adaptations [7],[8]. Given the instability and temporary nature of the resources, the microbial community must be able to exploit the available resources rapidly and efficiently [9]. It has been found that biological activity under the stones, as described by respiration and an increase in biomass, is prolonged [10]. Flint stones are sedimentary rocks with a hardness of 7 on the Mohs scale. They are cryptocrystalline, dark grey and translucent rocks and are known to play an important role in dew formation due to their distinct thermal properties under fluctuating temperatures. Despite the wide range of temperature fluctuation (in autumn, from a maximum of 72 °C to a minimum of 10 °C), cooling to the dew point results in the condensation of air moisture on flint stones at rates greater than on the surrounding surfaces, with practically no infiltration into the stone. The dew trickles down under the stones, where it triggers biological activity. In a Negev Desert ecosystem, dew is one of the most predictable sources of moisture. As the low temperatures climb along the slopes at night, the wide surface area of the stones acts as condensation surfaces, promoting the condensation of water vapors from the air [5]. Dew condenses on the stones for about 210 nights of the year [1],[5].

Nevertheless, even though this phenomenon has been long described, there is little information available on the impact of flint-stone cover on microbial community diversity and the associated functional diversity, as compared with the impact of the open spaces between the stones. This study aims to examine the potential effect of flint stones on microbial activity, and its functional and taxonomic diversity compared with the bare (control) soil. We hypothesized that the presence of flint stones affects microbial diversity, inducing a more homogeneous population than in the soils of the uncovered open space.

## 2. Materials and Method

### 2.1. Study site

Samples were collected at a site located in the northern Negev Desert highlands west of Yeruham, 7 km north of Sde Boker (30°55′N 34°47′E). This area is characterized by a temperate desert climate, with mild, rainy winters and hot, dry summers. The multiannual mean rainfall is about 90 mm, most of which occurs in scattered showers during the winter. The study site is a hill with a southwest slope, 1 km long and approximately 50 m high, at about 600 m above sea level. This site was chosen because of its abundance of flint stones and the high percentage of stones acting as a habitat for hypolithic algae.

**Figure 1.**
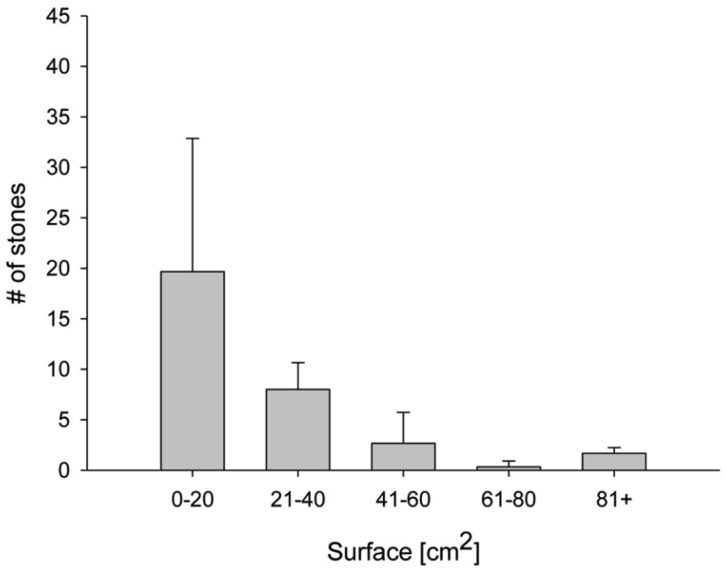
Changes in the mean number (n = 5) of the stones relative to their projection on the soil surface in an area of 1 m^2^.

The distribution of stones according to size is presented in Figure 1. A significant decrease in the number of stones as related to an increase in their size can be observed, where over 50% of the stones found were a maximum of 20 cm^2^ each.

### 2.2. Soil sampling

Soil samples were collected in August 2014 at the end of the summer season. Soil (2–3 mm depth) was sampled in four replicates collected under large flint stones. Each individual replicate was obtained by pooled samples collected under 25 stones. In addition, four control replicates were collected from the adjacent open spaces between flint stones. Each control sample was also obtained by pooling 25 samples; each sample represented an area of 2 × 2 cm and 2–3 mm depth (biocrust samples). Soil samples were placed in individual plastic bags and stored in cool, insulated boxes until arrival at the laboratory. In addition, four areas of 1 m^2^ each were chosen for estimating flint-stone surface cover via surface projection. At the laboratory, soil samples were sieved through a 2-mm sieve to remove stones, roots, and other organic debris, and then stored at 4 °C until used.

### 2.3. Laboratory analysis

(1) Soil moisture was determined gravimetrically by drying soil samples for 24 h at 105 °C, and was expressed as percentage of dry weight [11].

(2) Organic matter (OM) was determined by oxidation with 1 N potassium dichromate in an acidic medium, according to Rowell [12].

(3) Total soluble nitrogen (TSN) was determined by chemical extraction and color reactions using a Skalar AutoAnalyzer [13].

(4) CO_2_ evolution and microbial biomass were measured by dye plates—a colorimetric reaction using absorbent alkali with the ability to measure carbon dioxide evolution. Water (25 µl) was added to whole soil samples (0.3 g) in deep well plates covered by the dye plates in order to measure respiration. Glucose was added to determine microbial biomass according to the substrate-induced respiration method. CO_2_ values were measured after 2 h of soil respiration [14]. The last two mentioned were used for calculating the competition efficiency of the soil microbial population. The metabolic quotient (qCO_2_) was calculated according to the equation qCO_2_ = CO_2_ production/biomass. The metabolic quotient for CO_2_ is a specific parameter for evaluating the effects of environmental conditions on soil microbial biomass [15],[16].

**Table 1.** The different carbon sources added to soil in MicroResp™ divided into chemical categories.

Aromatic carboxylic acids	Amino acids	Carbohydrates	Carboxylic acids
3,4-Dihydroxybenzoic acid (protocatechuic acid)	L-Alanine Arginine L-Cysteine HCl g-Amino butyric acid L-Lysine N-Acetyl-glucosamine	L-Arabinose D-Fructose D-Galactose D-Glucose Trehalose	Citric acid L-Malic acid Oxalic acid

(5) Microbial functional diversity and community-level physiological profile (CLPP) were detected using the MicroResp™ plate method [17]. Fifteen different carbon sources of carbohydrates, carboxylic acids, amino acids, and aromatic carboxylic acids (Table 1) were added to whole soil samples (0.32 g in each well) in deep well plates. CO_2_ evolution was measured by dye plates (see section 4). The plates were read twice in a spectrophotometer at 590 nm, just before the plates were placed on the deep wells containing the soil samples (Time 0) and after 4 h of soil respiration (Time 1). During that time, the plates were incubated in the dark at 27 °C. The result for each well was calculated in comparison to the 16^th^ well, which contained the same soil sample and water, measuring the basal respiration with no carbon source at all.

(6) Microbial functional diversity was estimated using the Shannon-Weaver index [H' = –ΣPi (ln Pi)], where Pi is the ratio of the activity of a particular substrate [18].

(7) Viable bacterial counts—For viable counts, serial dilutions of solution containing 1 g soil and 9 ml water (double distilled) were prepared. An aliquot of 0.2 ml of the 10^−4^ dilution was plated on tryptic soy broth (TSB) agar. Plates were incubated at 27 °C for 7 days, and viable counts were determined as colony-forming units (CFUs) per 1 g dry soil.

(8) Viable fungal counts—For viable counts, serial dilutions of solution containing 1 g soil and 9 ml water (double distilled) were prepared. An aliquot of 0.2 ml of the 10^−2^ dilution was spotted on Rose-Bengal agar containing streptomycin (100 mg ml^−1^). Plates were incubated at 27 °C for 7 days, and viable counts were determined as colony-forming units (CFUs) per 1 g dry soil.

(9) DNA extraction and amplification

Genomic DNA from soil was extracted from each sample using the MO BIO PowerSoil™ DNA Isolation Kit (MO BIO Laboratories, Inc., Carlsbad, CA). The bacterial 16S rRNA gene and the fungal ITS1 region were amplified from the total DNA using FAM (6-carboxyfluorescein)-labeled polymerase chain reaction (PCR) forward primer and an unlabeled reverse primer. PCR amplification of the 16S rRNA gene was performed using a universal forward primer, as described by Muyzer and Ramsing [19], i.e., 341 (F), and a reverse primer derived from the conserved region between positions 683 and 707 [20], i.e., 700 (R). These primers flank the two variable regions, V3 and V4, of the 16S gene [20]. The reaction was carried out in a total volume of a 50 ml mix containing 2 µl DNA template, 0.5 µl Phusion® High-Fidelity DNA polymerase (New England BioLabs, M0530S), 1 mM MgCl_2_, 0.2 mM dNTPs, 10 µl 5× Phusion HF buffer, and 400 nM of each of the two primers. The thermal profile involved PCR amplification reaction, with initial denaturation for 30 s at 98 °C, followed by 35 cycles of 98 °C for 10 s, 60.6 °C for 15 s, 72 °C for 15 s, and 72 °C for 5-min final extension step.

For fungal-community analysis, the ITS1 region of ribosomal DNA was amplified with the primer pair, ITS1 (F) [21] and ITS2 (R) [22]. The PCR was performed for the ITS1 region in a total volume of 50 ml reaction mixure containing 2 µl DNA template, 0.5 µl Phusion® High-Fidelity DNA polymerase (New England BioLabs, M0530S), 1 mM MgCl_2_, 0.2 mM dNTPs, 10 µl 5× Phusion HF buffer, and 400 nM of each of the two primers. The thermal profile involved PCR amplification reaction with initial denaturation for 30 s at 98 °C, followed by 35 cycles of 98 °C for 10 s, 65 °C for 15 s, 72 °C for 10 s, and 72 °C for 5-min final extension step.

PCR product size was assessed on a 2% agarose gel (∼360 bp for the 16S rRNA, and ∼150–300 bp for the ITS1 region). DNA bands were extracted from gel using 3 volumes of 6 M NaI, and purified on Zymo-Spin™ IIN columns. Following purification, PCR amplicons were prepared for sequencing using the Ion Torrent Personal Genome Machine (PGN) (Life Technologies).

Sequencing: Libraries from each sample were prepared using the Ion Xpress™ Plus Fragment Library Kit (Life Technologies) with barcodes from the Ion Xpress Barcode Adapters 1-16 Kit (Life Technologies). The libraries were quantified and qualified using a DNA 1000 Bioanalyzer chip. The emulsion PCR was carried out on a OneTouch 2 system (Life Technologies) using the Ion PGM™ Template OT2 200 Kit (Life Technologies). The quality of the unenriched spheres was checked on a Qubit 2.0 using the Ion Sphere Quality Control Kit (Life Technologies). Sequencing of the amplicon libraries was carried out on the Ion Torrent Personal Genome Machine (PGM) system using the Ion Sequencing 200 Kit V2 (all Life Technologies), following the manufacturer's protocol.

(10) Taxonomic diversity analysis

The protocol described by Schloss et al. [23] was used to reduce the errors in the sequencing dataset and eliminate artifacts and chimeras [24]. For bacteria, sequences under 200 bp were eliminated [24]. Data were aligned on the mothur-supplied SILVA-based reference alignment [25], eventually followed by taxonomic assignment via a mothur-formatted version of the Ribosomal Database Project (RDP) training set [26]. The analysis produced 3295 bacterial operational taxonomical units (OTUs).

The same pipeline was used for the fungal database. The dataset was trimmed to eliminate any sequences shorter than 150 bp. However, taxonomic assignments were carried out using the “UNITE + INSD” vs.7 FASTA and taxonomy files [27]. The analysis produced 3182 fungal OTUs. The ITS1-based identification also classified 185 sequences as protozoa (Ciliophora).

(11) Abundance and richness estimates

Both Chao1 estimator and ACE index were calculated as implemented in mothur [24] and summarized as described by Hughes et al. [28].

## 3. Results

### 3.1. Soil moisture, organic matter, and TSN

**Figure 2.**
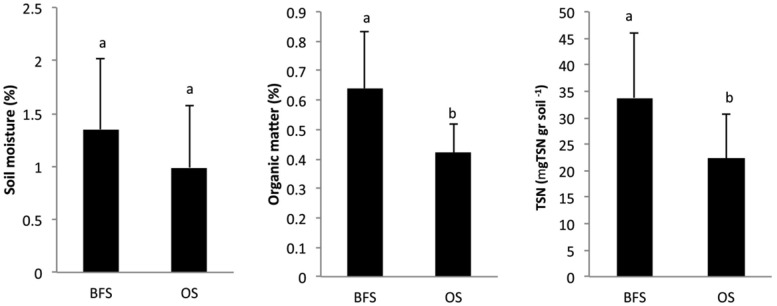
Changes in mean values (n = 4) of soil moisture at sampling (SM, %), organic matter (OM, %), and total soluble nitrogen (TSN, ppm) in soil samples collected beneath flint stones (BFS) or from the open spaces (OS) between the flint stones.

Approaching the end of the long, dry summer season, the total amount of rainfall becomes negligible and consequently, no significant differences (*P* > 0.05) were observed in the soil moisture levels (Figure 2). Nevertheless, the mean moisture levels at the time of sampling were found to be 1.35% for the BFS and 0.99% for the OS habitats. While this was not statistically significant, it might possibly suggest an effect of limited night dew contribution throughout the season. Significant (*P* < 0.05) differences between the two sampling locations were obtained for organic matter (%), with mean values of 0.64% for BFS and 0.42% for OS. The total soluble nitrogen (TSN) values were significantly higher (*P* < 0.05) for BFS [33.14 mg g^−1^ dry weight (dw) soil] in comparison with OS soil samples (21.90 mg g^−1^ dw soil) (Figure 2; Table 2).

### 3.2. Microbial respiration and microbial biomass

**Figure 3.**
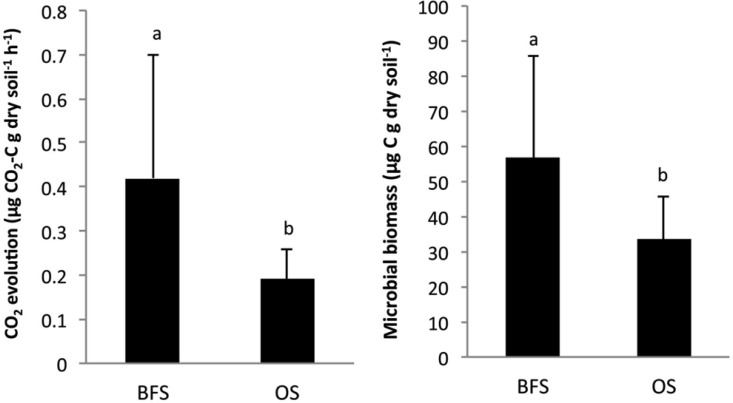
Changes in mean values (n = 4) of soil microbial CO_2_ evolution and microbial biomass in soil samples collected beneath flint stones (BFS) or from the open spaces (OS) between the flint stones.

The variability in microbial respiration (MR) rates and microbial biomass (MB) of soil samples taken between and under flint stones, is summarized in Figure 3. Both parameters were significantly (*P* < 0.05) higher for BFS, with an MR of 0.42 µg CO_2_-C g dry soil^−1^ h^−1^ and MB of 56.8 µg C g dry soil^−1^, than for the OS, where the MR reached only 0.19 42 µg CO_2_-C g dry soil^−1^ h^−1^ and MB was lower, at 33.6 µg C g dry soil^−1^. No significant relationships were observed between MR and MB (Table 3). It should also be mentioned here that the total soluble nitrogen (TSN; mg g^−1^ dw soil) did not show any significant correlation with either MB or MR (Figure 3).

**Figure 4.**
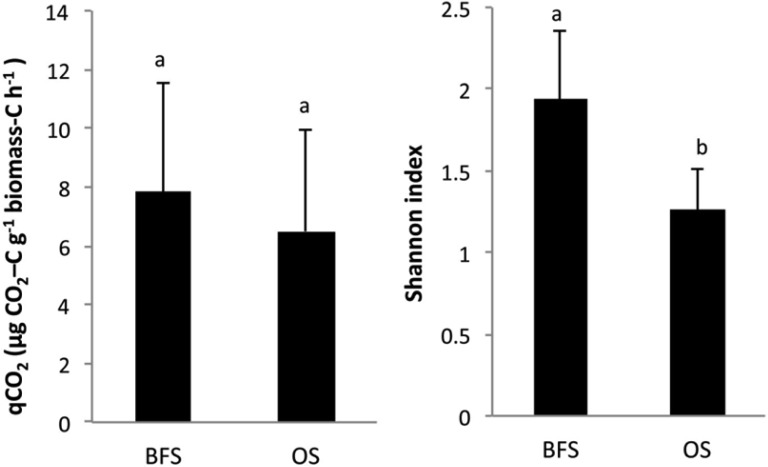
Changes in mean values (n = 4) of qCO_2_ and CLPP Shannon index values in soil samples collected beneath flint stones (BFS) or from the open spaces (OS) between the flint stones.

The metabolic quotient (qCO_2_) was not significantly different between the two habitats (Figure 4). However, microbial functional diversity (H') was found to be significantly (*P* < 0.001) greater in the BFS samples (Figure 4; Table 2).

**Figure 5.**
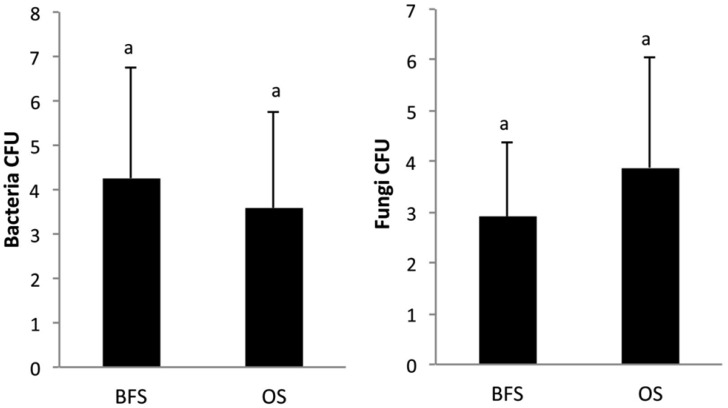
Changes in mean values (n = 4) of colony-forming units (CFUs) of bacterial and fungal communities in soil samples collected beneath flint stones (BFS) or from the open spaces (OS) between the flint stones. Values are expressed as 10^6^ CFU g^−1^ dry soil for bacteria and 10^3^ CFU g^−1^ dry soil for fungi.

The mean count of bacterial colony-forming units (CFUs) in the BFS samples was 4.26 × 10^6^ g^−1^ dry soil, and was not statistically significantly different from the 3.59 × 10^6^ CFU g^−1^ dry soil counts in the OS samples. For fungi, CFU values were 2.92 × 10^3^ g^−1^ dry soil and 3.88 × 10^3^ g^−1^ dry soil for the BFS and OS samples, respectively, without any significant differences between them (Figure 5; Table 2).

### 3.3. Community-level physiological profile (CLPP)

**Figure 6.**
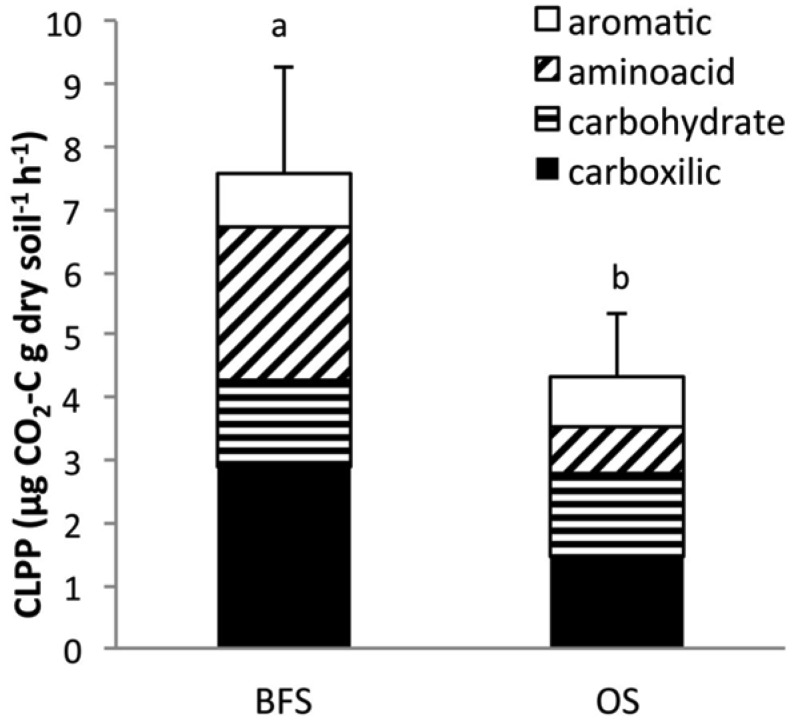
Substrate utilization profiles for soil samples collected beneath flint stones (BFS) or from the open spaces (OS) between the flint stones.

Of the 15 carbon substrates, as grouped in four carbon-substrate categories (aromatic acids, carboxylic acids, amino acids, and carbohydrates), only carboxylic acids and amino acids were found to play a significant role in CLPP variability (P = 0.008 and P = 0.002, respectively) in the two sampling locations. The CLPP values were significantly greater for BFS in comparison to OS (*P* < 0.05) (Figure 6).

For the BFS soil samples, the highest utilization levels were found for carboxylic and amino acids, at 2.92 and 2.43 µg CO_2_-C g^−1^ dry soil h^−1^, respectively. For the OS soil samples, the highest utilization values were measured for carboxylic acids, which were two-fold lower than the values found for the BFS samples, i.e., 1.46 µg CO_2_-C g^−1^ dry soil h^−1^, and for carbohydrates, i.e., 1.31 µg CO_2_-C g^−1^ dry soil h^−1^ consumption value. No significant differences in aromatic substrate utilization were observed.

### 3.4. Microbial diversity

**Figure 7.**
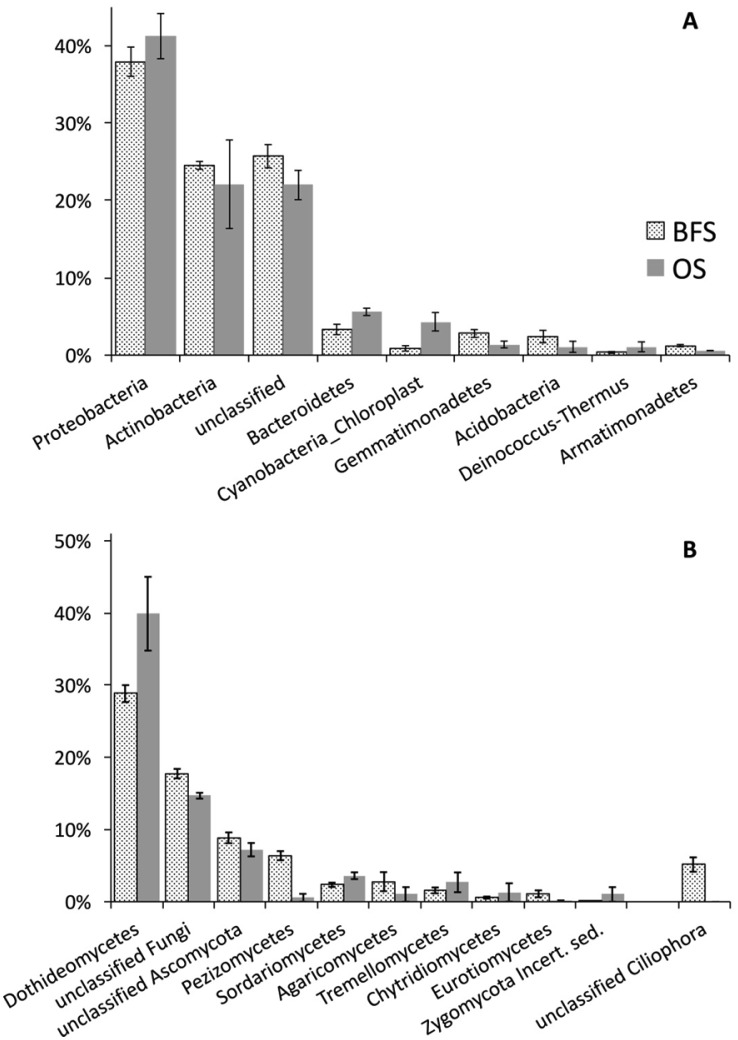
Taxonomic distribution of dominant bacterial phyla (A) and fungal classes (B) for samples collected beneath flint stones (BFS) or from the open spaces (OS). Error bars are the 95% confidence intervals.

Dominant bacterial and fungal taxa are summarized graphically in Figure 7. The graphs summarize 3276 bacterial OTUs and 2962 fungal OTUs. The ITS1 sequences, used for fungal diversity assessment, also identified the presence of protozoa (i.e., Ciliophora, 185 OTUs). Unclassified ITS sequences (979 OTUs) were not included. Bacterial phyla with abundance of less than 1% (Chloroflexi, Firmicutes, Nitrospira, and TM7) are not presented here; fungal classes with abundance of less than 1% are also not visualized here, but were included in the analyses. Sequencing recovery rates, as described by the mothur-calculated [21] coverage for the taxonomically resolved sequences, were good for both fungi and bacteria (Table 3).

### 3.5. Alpha diversity

Diversity indices were calculated in mothur on phylogenetic-identity-resolved datasets (Table 3). An abundance coverage estimator (ACE) that describes rare taxa as taxa represented by less than 10 OTUs, found no differences in fungal diversity. The Chao1 estimator, which assigns greater richness to data rich in singletons, shows a slight increase in diversity for BFS that is not statistically significant.

The inverse Simpson index, an index more sensitive to evenness than richness, confirms the ACE results while suggesting a relatively similar species evenness among the habitats. The trends are similar but more accentuated for bacteria, albeit not statistically significant. These results indicate similar alpha diversities for both habitats.

**Table 2.** Univariate analysis of variance (ANOVA) for soil properties and microbial activity in samples collected below flint stones and in the open space between stones (n = 4).

	F	*P* value
SM (%)	1.81	NS
OM (%)	4.63	0.05
TSN (mg g^−1^ soil)	7.11	0.01
MR (µg CO_2_-C g^−1^ dry soil h^−1^)	8.1	0.009
MB (µg C g dry soil^−1^)	6.55	0.02
*H'*	23.43	<0.0001
qCO_2_	0.86	NS
CFU bacteria	0.5	NS
CFU fungi	1.61	NS
MicroResp™, Carboxylic Acids (µg CO_2_-C g^−1^ dry soil h^−1^)	8.54	0.008
MicroResp™, Aromatic Acids (µg CO_2_-C g^−1^ dry soil h^−1^)	0.03	NS
MicroResp™, Carbohydrates (µg CO_2_-C g^−1^ dry soil h^−1^)	0.02	NS
MicroResp™, Aminoacids (µg CO_2_-C g^−1^ dry soil h^−1^)	13.96	0.002
CLPP	4.99	0.05

**Table 3.** Bacterial and fungal alpha diversity indices as calculated in mothur [24].

		Diversity indices (95% confidence intervals)			Coverage
		ACE	Inverse Simpson	Chao1	
Fungi	OS	43–87	5.4–9.2	34–72	92 ± 7%
	BFS	49–81	6.9–9.5	48–90	97 ± 2%
Bacteria	OS	88–143	9.3–12.5	70–122	95 ± 0.5%
	BFS	128–195	9.4–12.8	109–201	95 ± 0.5%

ACE, abundance coverage estimator.

### 3.6. Beta diversity

In general, there were little noticeable differences in the proportional abundance of bacterial phyla between the two habitats. Proteobacteria and Actinobacteria dominated both habitats at statistically equal proportions. This first observation does not seem support the hypothesis that flint stones modify the overall microbial diversity structure. However, a more detailed assessment of bacterial diversity based on an OTU alignment has shown that 81.1% of all OTUs were found in BFS, and only 50.2% were also found in the OS habitat (as calculated in SplitsTree4; [29]). The same analysis for fungi showed an even more dramatic difference, with only 5% of the OTUs in the OS habitat and 98% of the OTUs recovered in the BFS habitat. Close to a quarter of all bacterial OTUs could not be identified.

A detailed look at the fungal taxonomy indicated that 53.5% of the fungal genera in BFS (represented by 16.4% of all BFS OTUs) were unique to BFS; the unique OS genera were only 23.3% (represented by 3.2% of all OS OTUs) of all OS-identified genera. The same analysis for bacteria showed 28% of BFS genera to be unique to BFS (representing 5.5% of total OTUs) and 13% of OS genera were unique to OS (representing 1.6% of OTUs). These observations point to a common core of the microbial diversity across the BSF and OS locations, but with a significant distinct pattern of diversity among lower-abundance taxa. This indicates a general uniformity in the environmental conditions, probably expected at the end of the long, dry summer season, but with sufficiently distinct, likely long-term environmental particularities to allow for distinct taxonomic structure across the two locations, especially for the rarer taxonomic groups.

Thus, sequences for more stress-resistant bacterial taxa, such as Deinococcus-Thermus phylum, known to include radiation and desiccation resistant species [30] and the Thermomicrobia class of the Chloroflexi phylum [31], were found in a slightly larger proportion in the OS community. Cyanobacteria were also more obvious members of the OS communities (Figure 7). On the other hand, the BFS community was also well represented in taxa known to be adapted to low-moisture soils (e.g., Gemmatimonadetes [32]) or oligotrophic conditions (e.g., Armatimonadetes [33]). In general, common soil phyla or classes such as Acidobacteria, Betaproteobacteria, and Gammaproteobacteria made up a larger proportion of the BFS community. Bacteroidetes were proportionally slightly more abundant in the OS community [34].

As seen in Figure 7, BFS diversity was greater for operculate discomycetes (Pezizomycetes, mainly *Ascobolus* spp.), commonly associated with higher organic-matter soils supporting saprotrophs. While Dothideomycetes were proportionally more dominant in the OS, they are associated with species similar to *Alternaria* spp. On the other hand, other Dothideomycetes genera, e.g., Preussia, were found only in BFS. It is interesting to note that *Ascobolus* spp. and *Preussia* spp., both more likely to be found in the BFS, are commonly found in organic-rich substrates [35],[36]. On the other hand, *Alternaria* spp., which was more likely to be recovered in the OS, is a common plant pathogen, often surviving as spores resistant to dehydration and solar radiation. Protozoa (Ciliophora), widely distributed across climatic regions, including extreme deserts [37], were exclusively identified under the flint stones, suggesting their sensitivity to extreme desiccation and radiation. The autotroph groups (as described by the 16S rDNA Cyanobacteria-Chloroplast sequence assignments) seem to have been found in a larger proportion in the OS samples. One should note that this is probably nothing more than a reflection of the overall lower density of rDNA in the OS soils.

## 4. Discussion

Differences in organic matter are probably linked to minor differences in water availability, and play an important role in determining a microbial community [38],[39]. Being impermeable and resistant to weathering, the flint stone is found to lack endolithic lichens [40], and creates a higher amount of dew that penetrates below the flint stone in comparison to other substrates [41]. This, combined with protection from solar radiation, creates a less-extreme environment, as reflected in the biological activity. According to Belnap et al. [39], the diversity and abundance of soil microbial communities in arid regions vary with the amount of soil organic matter and with water availability; our findings show organic matter beneath the stones to be greater in comparison to that found in the open spaces. Diversity estimates have shown that saprobic organisms preferentially occur under the flint stone, confirming the role of organic carbon and water. Consequently, it is expected that the organic matter beneath the flint stones was one of the main factors affecting microbial CO_2_ evolution and biomass, most likely due to the activity of saprobic organisms. This was similar to the findings reported by Berner and Evenari [5], who worked on flint stones in the Negev Desert, and Clarck et al. [42], who reported on the response of a microbial community to water activity in the Chihuahuan Desert.

The less-extreme moisture and organic matter conditions under the flint stones also favored the presence of protists (Ciliophora) (known to occur where water films are also present). These protists are able to quickly respond to short-term water and resource availability, and possess efficient encysting capabilities [43]. Preussia fungi, previously reported as common endophytes of desert plants [44], were also unique for the BFS locations. The BFS genetic diversity was also matched by enhanced functional diversity, as measured via CLPP. While alpha diversity was statistically similar throughout the two habitats, fungal phylogenetic diversity was noticeably greater under the flint stones in comparison to the open spaces. Commonality in taxonomic diversity is a result of the location of the two habitats in close proximity. The diversity of the two habitats indicated that both desiccation and oligotrophy are common factors governing the function and diversity of the microbial communities. Nevertheless, differences in the density of fungal saprobes were a direct result of site-specific selection, probably under the transient distinctiveness of abiotic conditions.

The presence of saprotrophs, in conjunction with the observed greater proportional density of copiotrophic bacterial phyla such as Betaproteobacteria and Gammaproteobacteria [34], suggests a more resource-rich environment under BFS. On the other hand, as noted in the results, dessication-resistant phyla and oligotrophs are also present in the BFS habitats. The contradictions in the community functions that can be inferred from the distinct taxonomic profile are clearly a direct indication of the transient state of the abiotic conditions in BFS, favoring a diversification of the microbial populations. While resources as described by organic-matter concentrations are present in greater amounts in BFS, the transient characteristics of moisture [5] clearly govern the discontinuity between oligotrophy and copiotrophy, and also create survival conditions for taxa with variable stress resistance. On the other hand, some taxa found in the OS habitats suggested extreme stress resilience, including solar radiation stress, a stress mitigated by flint stone cover. A more detailed assessment of the functional resilience and its associated food-web structure induced by the stone cover is of interest for understanding biodiversity and ecological resilience under a changing climate.

## 5. Conclusions

Flint stones create unique biological hotspots in arid desert ecosystems that allow for the selection of a taxonomically and functionally complex microbial community. This diversity is directly linked to the transient characteristics of the governing abiotic factors. Consequently, a large number of organisms capable of a wider range of catabolic processes accumulate under the flint stones. This leads to enhanced carbon production, which enhances biological activities even more, in a feedback loop. While there is clear evidence of genetic fluxes from the flint-stone habitats to the open spaces, the extreme conditions in the open spaces did not show any unique resistant to taxa.
